# Enhancing the Mechanical and Rheological Properties of Coal-Based Geopolymer Grouting Materials with Nano-SiO_2_ and Polypropylene Fibers

**DOI:** 10.3390/polym17243338

**Published:** 2025-12-18

**Authors:** Sai Liu, Lei Zhang, Ning Hou, Wenxuan Meng

**Affiliations:** 1 National Energy Group Guoyuan Electric Power Co., Ltd., Beijing 100011, China; 20001540@ceic.com; 2 Shenhua Geol Explorat Co., Ltd., Beijing 100089, China; 18910962002@163.com; 3 School of Engineering and Technology, China University of Geosciences Beijing, Beijing 100083, China; 15065568720@163.com

**Keywords:** nano-SiO_2_, polypropylene fiber, coal-based solid waste, geopolymer, grouting material

## Abstract

In order to address the ineffective utilization of industrial solid wastes—particularly fly ash—under the “coal-power integration” model, and to improve the performance of coal-based solid waste geopolymer grouting materials (CBGWG) under dynamic water conditions, this study selected fly ash and coal gangue as the main raw materials to jointly prepare dynamic water grouting slurry. The effects of nano-SiO_2_ and polypropylene fibers (PPF) on gelation time, initial setting time, bleeding rate, apparent viscosity, compressive strength, and flexural strength were systematically investigated. The experimental results indicate that when the nano-SiO_2_ content was increased to 1%, the water separation rate decreased by 85.8%, viscosity increased by 17.5%, and both gelation time and initial setting time were reduced by 51.5% and 18.6%, respectively. At a nano-SiO_2_ content of 0.75% and a PPF dosage of 1.5%, the compressive strength and flexural strength increased by 43.3% and 53%, respectively. However, when the PPF dosage was further increased to 2%, fiber agglomeration occurred during mixing, impairing uniform dispersion. Nano-SiO_2_ predominantly enhanced the early stiffness of the consolidated body, while PPF significantly improved ductility, residual load-bearing capacity, and energy dissipation, albeit at the expense of some stiffness. These two modifiers exhibited complementary effects in improving the mechanical properties of the grouting material. The optimal dosages of nano-SiO_2_ and PPF were determined to be 0.75% and 1.5%, respectively, achieving the best balance between mechanical properties and workability.

## 1. Introduction

With the continuous increase in the depth and intensity of coal mining, the geological environment encountered in mines has become increasingly complex. In particular, in zones characterized by well-developed aquifers and fracture networks, water inrush and seepage under dynamic hydraulic conditions have emerged as critical challenges that restrict safe and efficient coal production [1,2]. In mine water prevention and control projects, grouting reinforcement therefore has been widely employed as an effective technique for mitigating such geological hazards [3,4,5].

However, implementing water-plugging grouting operations under dynamic water conditions not only requires the slurry to possess good pumpability and flowability, but also necessitates rapid setting, strong resistance to hydraulic scouring, as well as high stability and long-term durability of the hardened grouted body [6,7,8]. As a decisive factor influencing grouting effectiveness, the performance of grouting materials directly determines the success of water inrush control and sealing. Geopolymer grouting materials, as environmentally friendly binders that can fully or partially replace ordinary Portland cement, have attracted increasing attention due to their relatively simple preparation process, low cost, good stability, and excellent impermeability [9,10]. Meanwhile, the “coal–power integration” development model has become an important transformation pathway for coal mines, whereby coal-fired power plants are constructed in proximity to mining areas to realize on-site energy conversion and utilization. Nevertheless, due to the remote location of most mines, the industrial by-products of power plants—especially fly ash—often cannot be effectively locally [11]. Therefore, developing high-performance coal-based solid waste geopolymer grouting materials (CBGWG) using locally available resources not only helps to alleviate the storage pressure of coal-derived solid wastes, but also effectively reduces the grouting cost associated with mine water disaster control.

However, CBGWG generally suffer from several drawbacks, including significant volumetric shrinkage, low and slow-developing strength, and poor resistance to chemical and physical erosion [12,13]. Zhu et al. [14] reported that the incorporation of carbon fibers can markedly enhance the compressive and flexural strength of grouting materials. Hawreen et al. [15] investigated the reinforcing effect of carbon nanotubes on cement-based materials and found that carbon nanotubes can effectively improve the compressive strength of the matrix. Liang [16] demonstrated that the addition of polypropylene fibers (PPF) significantly improves the drying-shrinkage resistance of cementitious grouts and leads to notable enhancements in fracture toughness and impact resistance. Jin et al. [17] examined the influence of nano-SiO_2_ on the mechanical properties of geopolymer concrete under wet–thermal–chloride salt environments, and reported that incorporating 1.5% nano-SiO_2_ increased the compressive strength by up to 25.8%. Woo et al. [18] found that nano-SiO_2_ can promote the formation of calcium silicate hydrate in cement-based systems, thereby markedly enhancing material strength. These findings clearly indicate that nano-SiO_2_ contributes to improved strength, durability, and thermal stability of geopolymer materials, while fibers such as carbon fibers and PPF effectively enhance crack resistance and toughness.

At present, studies on the combined effect of nano-SiO_2_ and fibers mostly employ the more cost-effective PPF. Althoey F. et al. [19] incorporated nano-SiO_2_ and PPF into ultra-high-strength geopolymer concrete (UHS-GPC) and reported significant enhancements in compressive, tensile, and flexural strengths, as well as in the elastic modulus. Dheyaaldin et al. [20] added 1–2% nano-silica and 0.5–1% PPF to low-calcium fly ash/blast furnace slag alkali-activated mortars, and found that, in addition to improved mechanical performance, the water absorption of the material was reduced. Feng et al. [21] further demonstrated that, compared with geopolymer concrete (GPC) modified by a single admixture, the combined incorporation of nano-SiO_2_ and hybrid fibers markedly improved both the rheological parameters and the compressive strength of GPC. In summary, these related studies are primarily focused on ordinary geopolymer concrete or mortar systems, whose material characteristics and application scenarios differ substantially from those of CBGWG. Grouting materials are required to provide superior pumpability and controllable setting time, among other properties; therefore, the synergistic enhancement mechanisms of nano-SiO_2_ and PPF in CBGWG still require in-depth investigation.

Based on this, fly ash and coal gangue were selected as the main coal-based solid wastes to prepare CBGWG grouting slurry suitable for dynamic water conditions, and the effects of nano-SiO_2_ and PPF on the gelation time, initial setting time, bleeding rate, apparent viscosity, compressive strength, and flexural strength of CBGWG were systematically investigated. The results of this study elucidate the synergistic enhancement mechanisms of nano-SiO_2_ and PPF on the rheological and mechanical behavior of CBGWG, expand the pathways for high-value resource utilization of coal-based solid wastes, and provide a theoretical basis and technical support for the design and optimization of high-efficiency, cost-effective, and environmentally friendly grouting materials for dynamic water plugging in coal mine engineering.

## 2. Experiment

### 2.1. Raw Materials

The raw materials used in the experiments included cement, coal gangue powder, fly ash, water glass (sodium silicate), NaOH solution, nano-SiO_2_, PPF, and water. Coal gangue and fly ash were obtained from an open-pit coal mine in Heilongjiang Province, China. Coal gangue powder was prepared by crushing and grinding using a planetary ball mill at 300 r/min for 60 min, followed by sieving through a 0.045 mm (325 mesh) square-hole sieve. The chemical compositions and basic performance indices of coal gangue powder and fly ash are summarized in Table 1.

**Figure 1:**
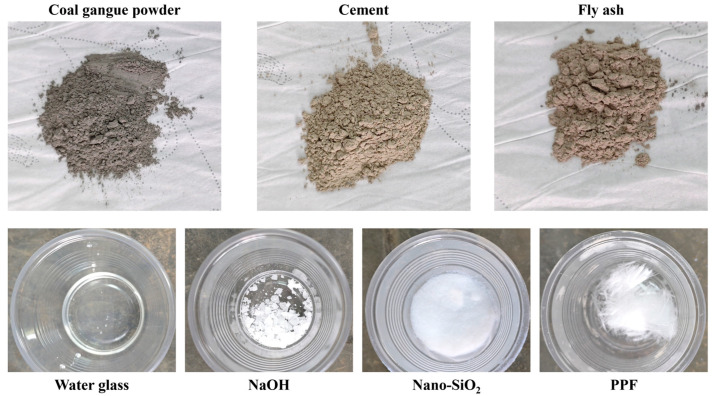
Raw material photos (PPF).

Sodium silicate was chosen as the quick-setting agent due to its stability and low cost, and was supplied by Luboshi Building Materials Co., Ltd. (Anhui, China), while solid NaOH flakes with a purity greater than 98%, supplied by Yongli Chemical Co., Ltd. (Tianjin, China) were used to adjust the modulus of the sodium silicate solution. The use of high-purity NaOH ensures accurate control of the OH^−^ concentration in the activator and minimizes the influence of impurities on the geopolymerization process. The detailed parameters of water glass are shown in Table 2. The cement employed was a 42.5 grade sulphoaluminate cement manufactured by Beijixiong Building Materials Co., Ltd. (Tangshan, China). The nano-SiO_2_ used in this study was supplied by Baisi Chemical Reagent Co., Ltd. (Zhongshan, China), and the PPF were supplied by Chuangsheng Building Materials Co., Ltd. (Shijiazhuang, China); the performance parameters of nano-SiO_2_ and PP fibers are provided in Table 3 and Table 4, respectively. All mixing water used in the experiments was ordinary tap water, and photographs of the raw materials are shown in Figure 1.

### 2.2. Experimental Design and Sample Preparation

In grouting applications under dynamic water conditions, the slurry must exhibit a short gelation time and rapid early strength development to resist washout, while maintaining a suitable viscosity to ensure smooth injection and effective penetration into fractures. At the same time, low bleeding and good stability are required to avoid segregation and to form a continuous, defect-free sealing body. These performance requirements guided the selection of the mix parameters in this study.

Based on the results of preliminary mix design tests and field construction experience, the water-to-binder ratio was set to 0.8 as a compromise that ensures adequate slurry flowability and pumpability. In addition, in order to provide sufficient alkalinity to effectively activate fly ash and coal gangue while avoiding an excessively rapid reaction, and considering that the time from slurry mixing to complete injection and diffusion in practical grouting operations should be at least 30 min, the modulus of the water glass was fixed at 1.4 according to the preliminary test results. The mix proportions of the slurry are listed in Table 5. First, nano-SiO_2_ was added at dosages of 0%, 0.25%, 0.5%, 0.75%, and 1% to evaluate its effects. Then, based on the optimal dosage of 0.75% nano-SiO_2_ (identified from the workability tests), PPF were incorporated at dosages of 0.5%, 1.0%, 1.5%, and 2.0% for further testing.

**Figure 2:**
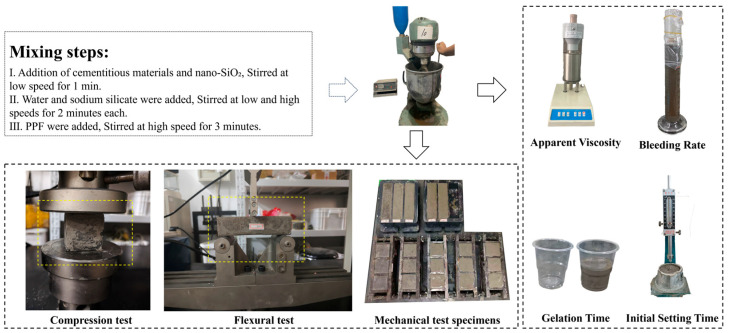
Flowchart of the experimental procedure.

The slurry was prepared using a cement paste mixer, following these steps: (1) the binder materials and nano-SiO_2_ were mixed slowly for 1 min; (2) water and water glass were added sequentially and mixed at low and high speeds for 2 min each; (3) PPF were gradually dispersed into the slurry and mixed for an additional 3 min. After mixing, part of the slurry was used for workability tests, while the remainder was cast into molds of 40 mm × 40 mm × 160 mm and 50 mm × 50 mm × 50 mm. After casting, the specimens were wrapped and sealed with plastic film to prevent moisture loss and stored in the laboratory at (20 ± 5) °C for 24 h before demolding. After demolding, they were cured in a standard curing chamber at a temperature of (20 ± 1) °C and a relative humidity of ≥95% until the designated testing ages. This curing regime was adopted to provide sufficient moisture and alkalinity for the dissolution of aluminosilicate phases in fly ash and coal gangue and for the continuous formation of the geopolymer gel, while at the same time approximating the temperature and humidity conditions encountered during in situ grouting in coal mines. In contrast to the elevated-temperature curing often used in laboratory geopolymer studies, the chosen curing conditions better reflect the practical service environment and thus allow a more realistic evaluation of the rheological and mechanical behavior of CBGWG. The Flowchart of the Experimental Procedure is shown in Figure 2.

### 2.3. Test Method

After slurry preparation, the following conventional performance tests were conducted: gelation time, initial setting time, bleeding rate, and apparent viscosity. Gelation time was measured using the inverted cup method, defined as the time when the slurry no longer flowed after the cup was tilted at 45°. Initial setting time was measured with a Vicat apparatus, defined as the time when the penetration needle sank into the paste and stopped at 4 mm ± 1 mm above the bottom plate. Bleeding rate was determined by placing 250 mL of slurry in a graduated cylinder and recording the volume of exuded water at 5 min intervals until stabilization, then calculating the ratio of exuded water. The apparent viscosity was determined using a six-speed rotational viscometer and was taken as one half of the stable reading at 600 r/min.

The compressive strength tests were conducted in accordance with the Chinese standard <Standard for test method of performance on building mortar> (JGJ/T 70-2009) [22], using a YAW-300B microcomputer-controlled testing machine (Jinan Zhong Luchang Testing Machine Manufacturing Co., Ltd., Jinan, China). Cube specimens with dimensions of 50 mm × 50 mm × 50 mm were tested under a displacement-controlled loading rate of 5 mm/min. Flexural strength tests were carried out with reference to <Norm for fracture test of hydraulic concrete> (DL/T 5332-2005) [23], using a 100 kN microcomputer-controlled electro-hydraulic servo universal testing machine. Prismatic specimens measuring 40 mm × 40 mm × 160 mm were tested in three-point bending with a span length of 120 mm and a loading rate of 0.1 mm/min. For both compressive and flexural tests, three specimens were tested in each group, and the arithmetic mean value was taken as the final test result. Photographs of the mechanical test setups are shown in Figure 2. The flexural strength was calculated using the following equation:

$$\sigma =\frac{1.5PL}{b^{3}}$$

where *σ* is the flexural strength of the specimen (MPa), *P* is the applied load (N), *L* is the spacing between two supports (mm), and *b* is the width of the prismatic square section (mm).

## 3. Results and Discussion

### 3.1. Workability of the Slurry

#### 3.1.1. Bleeding Rate

**Figure 3:**
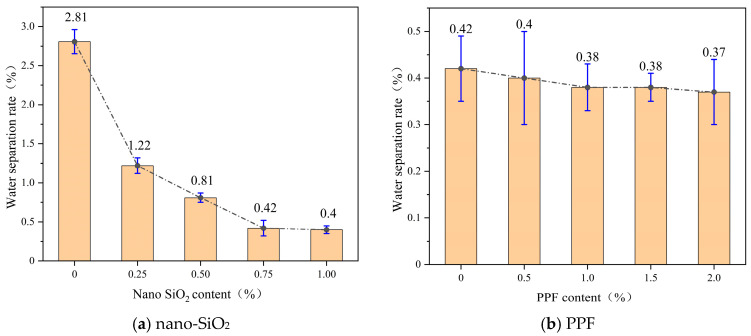
Water separation rate of slurry at different nano-SiO_2_ and PPF content levels. (**a**) nano-SiO_2_. (**b**) PPF.

Figure 3 presents the bleeding rate of the slurry with varying dosages of nano-SiO_2_ and PPF. It can be seen that the bleeding rate decreases as the contents of nano-SiO_2_ and PPF increase, showing a negative correlation. When the nano-SiO_2_ content increased from 0% to 1%, the bleeding rate dropped by 85.8%, with the rate of decline gradually slowing down. This is attributed to the ultra-high specific surface area and strong water absorption of nano-SiO_2_, which significantly improves the compactness of the slurry [24]. Its surface silanol groups react with hydration products, rapidly consuming free water and suppressing particle sedimentation and the formation of upward water channels. As the dosage increases, however, adsorption and nucleation sites tend to saturate, particle agglomeration intensifies, and the marginal densification effect weakens, leading to a reduced rate of bleeding decline [25].

In contrast, PPF are hydrophobic and inert materials. When the dosage increased from 0.5% to 2.0%, the bleeding rate decreased by only 7.5%. This is mainly because PPF enhance the viscous resistance of the slurry through physical interlocking, while their ability to chemically bind free water and block capillary channels is limited; therefore, their effect on reducing the bleeding rate is negligible.

#### 3.1.2. Apparent Viscosity

**Figure 4:**
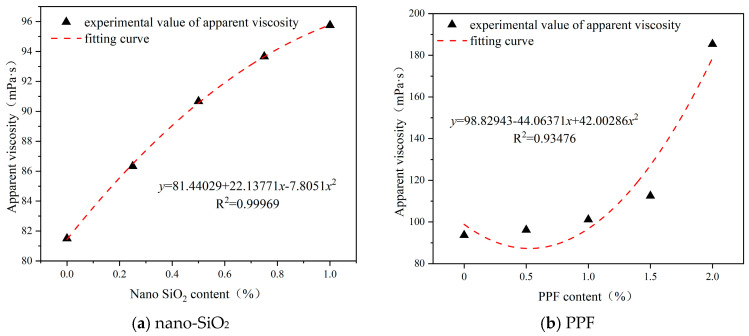
Apparent viscosity of slurry at different nano-SiO_2_ and PP fiber content levels. (**a**) nano-SiO_2_. (**b**) PPF.

Figure 4 shows the variation in apparent viscosity with different dosages of nano-SiO_2_ and PPF. The apparent viscosity of the slurry increased with increasing dosages of both modifiers, following a quadratic polynomial distribution. With the increase in nano-SiO_2_ dosage, the apparent viscosity of the slurry exhibited a trend of rapid initial growth followed by a gradual plateau, with the growth rate progressively decreasing. Specifically, when the dosage increased to 0.25%, 0.50%, 0.75%, and 1.0%, the apparent viscosity increased by 5.9%, 5.0%, 3.2%, and 2.2%, respectively. This nonlinear variation can be attributed to the combined effects of particle surface activity, water adsorption, and agglomeration phenomena at different dosage levels. At low dosage levels, nano-SiO_2_ particles possess an extremely high specific surface area and surface reactivity, enabling them to strongly interact with water molecules and ions within the slurry system. These interactions result in pronounced water-absorption effects and flocculation behavior [26]. Consequently, the internal friction between particles within the slurry is markedly increased, which significantly reduces fluidity and produces a sharp rise in apparent viscosity. Thus, the viscosity increment is most pronounced when a small amount of nano-SiO_2_ is incorporated.

Previous studies have shown that in cement-based slurries, due to the large specific surface area and high surface energy of nano-SiO_2_, it tends to agglomerate at higher dosages. The agglomerated nano-SiO_2_ particles cannot be used as fillers to fill the cement particles and instead surround the free water, which leads to an increase in the viscosity and a decrease in the flowability of cement-based materials [27,28,29]. Therefore, in this study, although the incorporation of nano-SiO_2_ leads to an increase in viscosity, it is likely that due to the agglomeration of nano-SiO_2_ particles and the gradual saturation of adsorption sites, the rate of increase in apparent viscosity gradually decreases as the dosage of nano-SiO_2_ increases.

The rate of increase in apparent viscosity gradually rose with increasing PPF dosage. When the fiber content was increased to 0.5%, 1.0%, 1.5%, and 2.0%, the apparent viscosity increased by 2.6%, 5.2%, 11.2%, and 64.8%, respectively. This behavior is closely related to the spatial distribution and interaction mechanisms of the fibers within the slurry matrix. At low dosages, the number of fibers is limited, and the extent of contact, overlapping, and interlocking among fibers is insufficient to form a stable skeleton structure. As a result, the influence on slurry viscosity is relatively small, and the flow of free water remains largely unrestricted. Under these conditions, the rheological behavior of the slurry is not substantially different from that of the fiber-free system [30].

As the dosage increases, however, the number of fibers within the slurry rises, and interactions between them gradually become more pronounced. Overlapping, entanglement, and interweaving effects lead to the formation of an incipient three-dimensional network structure. On the one hand, this structure significantly increases the resistance to flow, restricting the movement of solid particles; on the other hand, fibers help to redistribute and confine particles, thereby increasing the yield stress of the slurry and resulting in a more viscous system. At high dosages (>1.5%), the interweaving and accumulation of fibers become much more significant, and the three-dimensional network structure approaches completion. Fibers not only hinder the movement of free water but also strongly couple with slurry particles, forming a dense and highly constrained system. This combination of effects causes slurry fluidity to drop sharply, leading to a dramatic increase in apparent viscosity. Moreover, during mixing, noticeable fiber entanglement and agglomeration occurred at the 2.0% dosage, making it difficult to achieve a uniform dispersion. Such aggregation would hinder slurry pumpability and impede its effective propagation within the formation during practical field applications.

#### 3.1.3. Gelation Time and Initial Setting Time

**Figure 5:**
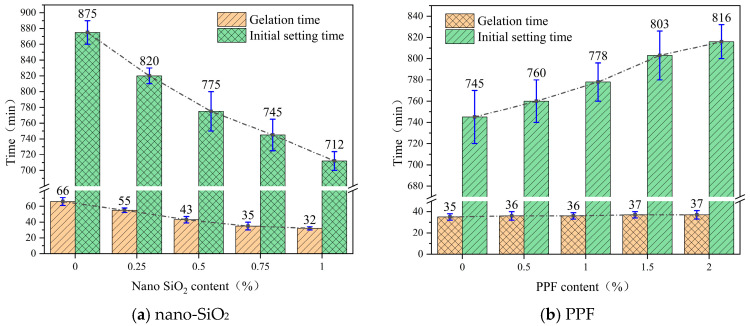
Gelation time and initial setting time of slurry at different nano-SiO_2_ and PP fiber content levels. (**a**) nano-SiO_2_. (**b**) PPF.

Figure 5 presents the variation curves of gelation time and initial setting time of the slurry under different dosages of nano-SiO_2_ and PPF. As can be observed, both gelation time and initial setting time decreased with increasing nano-SiO_2_ content, whereas they increased with higher PPF content. Specifically, when the dosage of nano-SiO_2_ was raised from 0% to 1.0%, gelation time and initial setting time were reduced by 51.5% and 18.6%, respectively, indicating that nano-SiO_2_ had a more pronounced effect on gelation time than on initial setting time. This phenomenon is not only attributed to the adsorption of free water by the hydroxyl groups on the surfaces of nano-SiO_2_ particles and their provision of nucleation sites for hydration products [31]. More importantly, the highly reactive silanol groups on nano-SiO_2_ can react with dissolved Al^3+^ and Ca^2+^ in the system, promoting the formation of N-A-S-H (sodium aluminosilicate hydrate) or C-A-S-H (calcium aluminosilicate hydrate) gels [32]. These gels function as the primary binding phases during the early coagulation stage, and their rapid nucleation markedly accelerates the establishment of the initial gel network. Consequently, the incorporation of nano-SiO_2_ drives the transition of the slurry from a sol state to a gel state, resulting in a substantial reduction in gelation time. Gelation time corresponds to the onset of microstructural connectivity within the slurry and is therefore highly sensitive to nanoscale nucleation and gel formation. In contrast, initial setting time reflects the development of macroscopic mechanical strength, which requires more extensive gel densification and structural stabilization, and is thus less affected.

As the PPF dosage increased from 0% to 2.0%, the gelation time and initial setting time were prolonged by 5.7% and 9.5%, respectively, with a more pronounced effect observed on gelation time. This behavior is closely related to the fact that PPF are chemically inert within the slurry system. Unlike nano-SiO_2_, PPF do not participate in chemical reactions, nor do they possess the ability to chemically bind free water. Instead, they primarily alter the rheological behavior of the slurry through physical effects. When incorporated at a certain dosage, PPF introduce spatial separation and dilution effects in the slurry. On the one hand, the presence of fibers hinders direct contact between particles, thereby weakening the bridging and connectivity of hydration products. On the other hand, the fiber surfaces become coated with a thin water film, which restricts the migration and diffusion of ions across the fiber–slurry interface. These combined effects slow down the hydration process, ultimately delaying the transformation of the slurry from a sol state to a gel state.

Because gelation time characterizes the early transition from a fluid slurry to a weak gel, it is more sensitive to the dilution and spatial isolation effects introduced by PPF, and thus increases more than the initial setting time. In contrast, the initial setting time is governed by the later-stage accumulation of hydration products and structural densification, and is therefore less affected by fibers. Overall, excessive PPF content delays microstructural connectivity and leads to unduly long setting times, which may reduce the efficiency and effectiveness of grouting operations.

### 3.2. Mechanical Properties of the Hardened Body

#### 3.2.1. Compressive Strength

**Figure 6:**
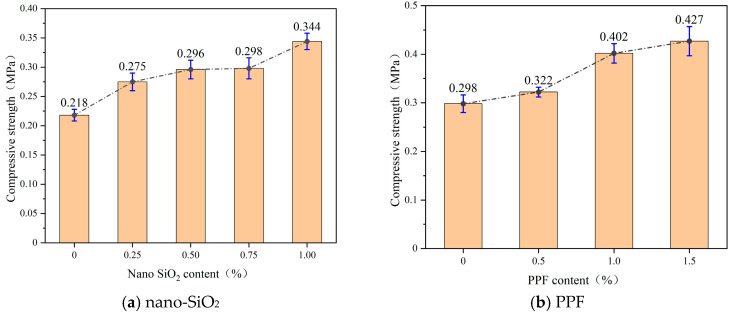
Compressive strength at different nano-SiO_2_ and PP fiber content levels. (**a**) nano-SiO_2_. (**b**) PPF.

Figure 6 shows the compressive strength of hardened slurry specimens at 7 days with varying dosages of nano-SiO_2_ and PPF. Both modifiers enhanced compressive strength, but the growth trend gradually leveled off with higher dosages. At low dosages, nano-SiO_2_ significantly improved strength due to its strong dispersibility and reinforcement effect on the internal structure of the matrix. However, with further increase, saturation effects reduced its strengthening efficiency. For PPF, compressive strength increased from 0.3 MPa to 0.43 MPa as the dosage rose from 0% to 1.5%, with the most significant improvement occurring at 0.5–1.0%. Excessive fiber dosage showed a diminishing strengthening effect.

**Figure 7:**
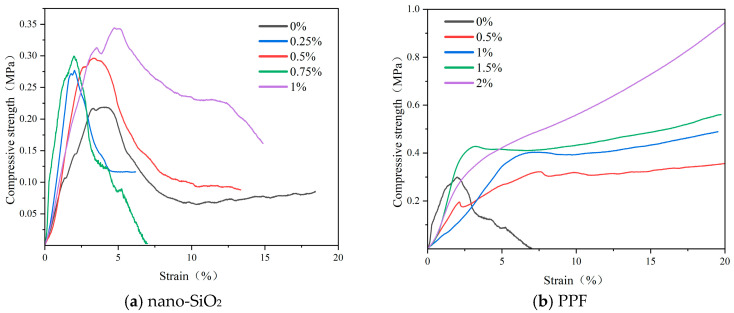
Stress–strain curves at different nano-SiO_2_ and PPF content levels. (**a**) nano-SiO_2_. (**b**) PPF.

**Figure 8:**
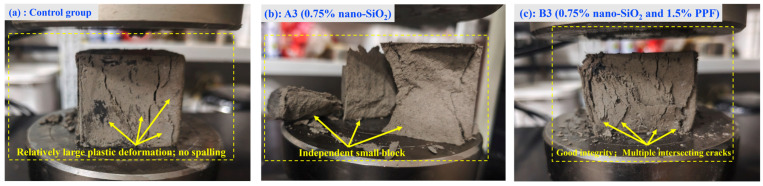
Failure Characteristics of Typical Compression Test Specimens. (**a**) Control Group (**b**) A3 (0.75% nano-SiO_2_) (**c**) B3 (0.75% nano-SiO_2_ and 1.5% PPF).

Figure 7 presents the stress–strain curves of the hardened bodies. With nano-SiO_2_, the initial slope increased significantly, indicating improved elastic modulus and early load-bearing capacity. However, failure remained brittle, with a rapid post-peak decline. At a dosage of 1.0%, the post-peak descent became slightly more moderate. As shown in Figure 8b, the compressive specimens incorporating nano-SiO_2_ exhibited a more pronounced brittle failure pattern compared to the control specimens in Figure 8a. This behavior is primarily attributed to the fact that nano-SiO_2_ refines the pore structure and promotes the formation of rigid gel phases, thereby enhancing the peak strength of the matrix [33]. However, it does not fundamentally alter the crack-propagation mode; once a dominant crack develops, the matrix still fails in a brittle manner.

In contrast, PPF reduced the initial slope, slightly lowering the stiffness but enhancing the deformability. Post-peak, the curves with PPF maintained higher residual stresses or even increased with strain, reflecting enhanced ductility, residual strength, and toughness. This post-peak behavior is governed by fiber–matrix interfacial mechanisms: PP fibers bridge developing cracks and transfer tensile stresses across the crack faces through interfacial bonding, mechanical interlocking, and frictional resistance [34]. As loading continues, progressive interfacial debonding and fiber pull-out dissipate considerable energy, delay crack localization, and result in a flatter, more ductile post-peak response [35]. At a fiber content of 2.0%, no distinct peak was observed, as the fibers sustained the load continuously. In this case, the dense fiber network forms multiple bridging points, suppresses the formation of a single dominant macrocrack and leading to a quasi–strain-hardening response at the material scale. Correspondingly, the failed specimens with PPF exhibited multiple intersecting cracks, while the broken fragments remained mechanically linked by the fibers rather than completely separating, indicating effective crack-bridging and pull-out of PP fibers during failure, as illustrated in Figure 8c.

The area under the stress–strain curves reflects energy absorption. Both nano-SiO_2_ and PPF significantly increased energy dissipation, enhancing impact and crack resistance. Overall, nano-SiO_2_ mainly improves stiffness and peak strength, whereas PPF enhance ductility and toughness. Their combination yields complementary effects on mechanical performance.

#### 3.2.2. Flexural Strength

**Figure 9:**
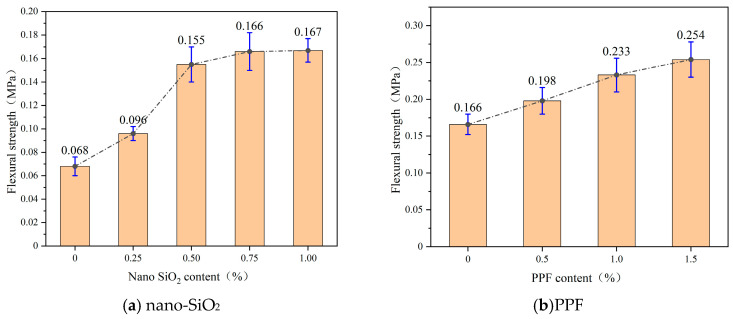
Flexural strength at different nano-SiO_2_ and PP fiber content levels. (**a**) nano-SiO_2_. (**b**) PPF.

Figure 9 presents the 7-day flexural strength of the consolidated slurry with varying dosages of nano-SiO_2_ and PP fibers. As shown, both nano-SiO_2_ and PPF improve flexural strength, with nano-SiO_2_ exhibiting a characteristic increase followed by a plateau. At low dosages (0% to 0.5%), nano-SiO_2_ significantly enhances flexural strength, with improvements of 41.0% and 61.5%, respectively. This enhancement is attributed to the refinement of the pore structure and the formation of rigid N-A-S-H or C-A-S-H gels at the matrix–aggregate interface, which improve stress transfer and crack resistance. However, as the dosage exceeds 0.75%, further improvements diminish due to the saturation of available interfacial defects and the risk of particle agglomeration, which can create local stress concentrations and reduce interface continuity. This leads to a flattening of the flexural strength curve.

**Figure 10:**
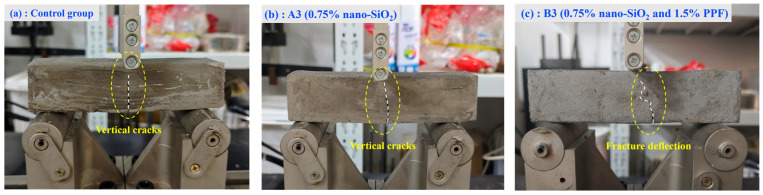
Failure Characteristics of Typical Flexural Test Specimens. (**a**) Control Group (**b**) A3 (0.75% nano-SiO_2_) (**c**) B3 (0.75% nano-SiO_2_ and 1.5% PPF).

As shown in Figure 10b, both the nano-SiO_2_-containing specimens and the control group in Figure 10a exhibited characteristic vertical fracture patterns. Although the addition of nano-SiO_2_ increased the strength, the fracture mode remained unchanged, primarily due to the material’s inherent brittleness, which caused vertical cracking under load.

The addition of PP fibers results in a near-linear increase in flexural strength with fiber content, improving by 53.0% from 0% to 1.5%. This improvement is primarily attributed to the fibers’ mechanical bridging effect and energy dissipation during pull-out, where fibers span cracks, carry part of the tensile stress, and prevent crack propagation [36]. As fiber content increases, more fibers participate in bridging and pull-out, leading to an almost linear rise in strength. Additionally, fibers promote crack deflection and branching, forcing cracks to deviate, bifurcate, or circumvent the fibers, thereby increasing the crack propagation path and the energy required for fracture. Correspondingly, as shown in Figure 10c, the fracture surfaces of PPF-modified specimens exhibit characteristic slanted crack paths, rather than vertical fractures, indicating that the fibers effectively promote crack deflection and branching, which enhances the material’s toughness.

However, it should be noted that PPF are chemically inert and do not participate in hydration or matrix densification like nano-SiO_2_. Their strengthening effect is purely physical, arising from their distribution and interaction with cracks. At higher fiber dosages (1.0–1.5%), uniform distribution ensures optimal crack bridging and deflection, achieving a stable and significant improvement in flexural performance. When fiber content exceeds this threshold, such as at 2.0%, issues like fiber clustering and entanglement can occur during mixing, making it difficult to achieve uniform dispersion. This negatively affects the slurry’s workability, and as a result, flexural strength testing was not conducted for this dosage.

### 3.3. Comprehensive Performance Evaluation and Engineering Implications

Based on the comprehensive results of slurry workability and consolidated body mechanical performance experiments, the water separation rate and gelation time were nearly the same at nano-SiO_2_ dosages of 0.75% and 1%, and the apparent viscosity, initial setting time, and flexural strength showed only minor differences. Considering the cost factors, the optimal dosage of nano-SiO_2_ is 0.75%.

For the consolidated body mechanical performance, when the nano-SiO_2_ dosage is 0.75%, a 2% dosage of PPF performs relatively well. However, compared to the 1.5% PPF dosage, the differences in water separation rate, gelation time, and initial setting time are minimal, but the viscosity increases by approximately 50.0%. Moreover, at 2.0% PPF, fiber entanglement and agglomeration occurred during mixing, making it difficult to achieve uniform dispersion, which would negatively affect slurry pumpability and diffusion in the formation during practical construction. Therefore, from a comprehensive perspective, the optimal dosage of nano-SiO_2_ is 0.75%, and the optimal dosage of PPF is 1.5%. The performance indicators of the CBGWG slurry with optimal dosages of nano-SiO_2_ and PPF are shown in Table 6. The key performance parameters meet the requirements for most dynamic water grouting applications in coal mines.

Incorporating nano-SiO_2_ and PPF into slurry formulations can have several environmental implications during the grouting construction process. PP fibers, being chemically inert and hydrophobic, do not participate in hydration reactions, and while they do not release harmful substances, their disposal at the end of the grouting operation can pose environmental concerns. PP fibers are non-biodegradable, and their accumulation in landfills can contribute to microplastic pollution. During the grouting process, dust generation from both nano-SiO_2_ and PP fibers must be controlled. If nano-SiO_2_ is used in the slurry, fine particles can become airborne during mixing and pumping, which may impact air quality if proper containment measures are not taken. Effective dust suppression and fiber dispersion techniques should be adopted to minimize these environmental impacts. Additionally, the uniform distribution of fibers in the slurry is crucial to avoid issues such as fiber entanglement or clustering, which can reduce slurry workability and affect its performance in the field. Therefore, careful management of dust control, fiber dispersion, and waste disposal practices is necessary to reduce the environmental footprint of the grouting process.

When scaling up the use of nano-SiO_2_ and PPF in slurry formulations for field applications, several challenges must be addressed. One of the primary issues is achieving uniform distribution of nano-SiO_2_ and PPF in large-scale mixing processes. In industrial-scale production, ensuring a consistent dispersion of these materials may require advanced mixing technologies or specialized equipment to prevent clumping and ensure optimal performance. Field implementation strategies should focus on optimizing the ratio of nano-SiO_2_ and PPF for different geological conditions to enhance the workability, pumpability, and sealing efficiency of the slurry. Furthermore, the environmental footprint associated with the large-scale production and disposal of these materials should be considered when planning their use in grouting applications, potentially integrating sustainable practices such as recycling or reusing waste materials.

## 4. Conclusions

(1) The water separation rate and apparent viscosity of the slurry both exhibit a negative correlation with the dosages of nano-SiO_2_ and PPF, with the apparent viscosity following a quadratic polynomial distribution pattern. As the dosage of nano-SiO_2_ increases, the rate of decrease in water separation and the rate of increase in apparent viscosity gradually diminish. The dosage of PPF has a relatively small effect on the water separation rate. However, when the PPF content reaches 2.0%, there is a sharp increase in apparent viscosity, accompanied by issues of fiber entanglement and agglomeration during mixing.
(2) Both the gelation time and initial setting time decreased with higher nano-SiO_2_ dosages but increased with higher PPF dosages. As the nano-SiO_2_ content increased from 0% to 1.0%, the gelation and initial setting times decreased by 51.5% and 18.6%, respectively. In contrast, as PPF content increased from 0% to 2.0%, the gelation and initial setting times increased by 5.7% and 9.5%, respectively, indicating that PP fibers had only a minor effect on setting behavior.
(3) The compressive and flexural strengths of the hardened slurry increased with the addition of both nano-SiO_2_ and PPF. For nano-SiO_2_, the strength improvement trend gradually diminished with higher dosages. For PPF, the flexural strength exhibited an approximately linear increase with dosage.
(4) Nano-SiO_2_ significantly enhanced the initial slope of the stress–strain curve, reflecting improved early stiffness, but failure still displayed a brittle mode. PPF, on the other hand, reduced the initial slope but improved ductility, residual strength, and toughness, with post-peak stresses either maintained or slightly increased. The combined addition of nano-SiO_2_ and PPF yielded complementary improvements in the mechanical properties of the consolidated body. Overall, the optimal dosages were determined to be 0.75% for nano-SiO_2_ and 1.5% for PPF. These results provide valuable insights for optimizing injection materials for dynamic water sealing in coal mines.

## Figures and Tables

**Table 1:** Chemical composition and performance indicators of coal gangue powder and fly ash.

Composition	Chemical Composition (Mass Fraction, %)								Density/(g/cm^3^)	Specific Surface Area/(g/cm^3^)	Fineness/%
	${SiO}_{2}$	${Al}_{2}O_{3}$	${Fe}_{2}O_{3}$	CaO	${Fe}_{2}O$	${TiO}_{2}$	${SO}_{3}$	C			
coal gangue powder	57.20	26.10	5.68	3.53	/	/	0.39	/	2.61	913	/
Fly ash	62.54	16.05	4.30	3.96	1.42	0.38	0.36	8.69	/	/	30

**Table 2:** Parameters of water glass.

$\omega \;({Na}_{2}O)$/%	$\omega \;({SiO}_{2})$/%	Modulus	Concentration/°Bé
8.30	26.20	3.20	40.00

**Table 3:** Basic performance parameters of nano-SiO_2_.

Tapped Density/(g/L)	Purity/%	Specific Surface Area/(m^2^/g)	Particle Size/nm	Suspension pH Value	Loss on Ignition/%
40.00~60.00	99.80	214.00	20.00	4.10	2.00

**Table 4:** Basic performance parameters of PPF.

Density/(g/cm^3^)	Length/mm	Equivalent Diameter/μm	Tensile Strength/MPa	Elastic Modulus/GPa	Elongation at Break/%
0.91	6.00	39.00	390.00	5.30	20.00

**Table 5:** Trial mix design plan (Fly ash:Coal gangue:Cement = 5:3:2. mass fraction, %).

Group	Nano-SiO_2_/%	PPF/%
Control group	0	0
A1	0.25	0
A2	0.5	0
A3	0.75	0
A4	1	0
B1	0.75	0.5
B2		1
B3		1.5
B4		2

**Table 6:** Performance of CBGWG at the optimal nano-SiO_2_ and PPF contents.

Bleeding Rate/%	Apparent Viscosity/mPa·s	Gelation Time/min	Initial Setting Time/min	7d Compressive Strength/MPa	7d Flexural Strength/MPa
0.38	112	37	816	0.42	0.25

## Data Availability

The original contributions presented in this study are included in the article. Further inquiries can be directed to the corresponding author.
